# Humoral Immune Response to AAV

**DOI:** 10.3389/fimmu.2013.00341

**Published:** 2013-10-18

**Authors:** Roberto Calcedo, James M. Wilson

**Affiliations:** ^1^Gene Therapy Program, Department of Pathology and Laboratory Medicine, University of Pennsylvania, Philadelphia, PA, USA

**Keywords:** adeno-associated virus, AAV, neutralizing antibody, immune response, gene therapy

## Abstract

Adeno-associated virus (AAV) is a member of the family *Parvoviridae* that has been widely used as a vector for gene therapy because of its safety profile, its ability to transduce both dividing and non-dividing cells, and its low immunogenicity. AAV has been detected in many different tissues of several animal species but has not been associated with any disease. As a result of natural infections, antibodies to AAV can be found in many animals including humans. It has been shown that pre-existing AAV antibodies can modulate the safety and efficacy of AAV vector-mediated gene therapy by blocking vector transduction or by redirecting distribution of AAV vectors to tissues other than the target organ. This review will summarize antibody responses against natural AAV infections, as well as AAV gene therapy vectors and their impact in the clinical development of AAV vectors for gene therapy. We will also review and discuss the various methods used for AAV antibody detection and strategies to overcome neutralizing antibodies in AAV-mediated gene therapy.

## Introduction

Adeno-associated virus (AAVs) is a member of the family *Parvoviridae* that has been widely used as a vector for gene therapy because of its safety profile, its ability to transduce both dividing and non-dividing cells, and its low immunogenicity. AAV is a small, non-enveloped single-stranded DNA virus that has been detected in many different tissues of several animal species (1, 2) but has not been associated with any disease (3, 4). In the past decade the discovery and development of new AAV types with dramatically improved *in vivo* performance and with unique seroreactivity and tissue tropisms (5–9) has situated AAV in the forefront of vector development for gene therapy trials. One of the most important aspects of the development of AAV as a clinical product is the impact of the host humoral immune response against its capsid. Several studies have shown that the induction of antibodies by natural exposure to AAV early in life can compromise the subsequent use of AAV as a gene therapy vector (10–14). Moreover administration of an AAV vector induces a potent and long term humoral response to AAV that may compromise the use of the same vector if a second administration is required (15, 16). Humoral immune responses to AAV can be of two types: neutralizing or binding (non-neutralizing). Neutralizing antibodies (NAbs) bind to AAV and through several mechanisms (17) inhibit AAV transduction of target cells. Non-NAbs bind to AAV and “flag” the virus without blocking AAV transduction. AAV NAbs have been the focus of many studies because of their significant deleterious effect on the efficacy of AAV-mediated gene therapy. Recent studies have shown that AAV binding antibodies may also have an impact on AAV vector distribution and safety (18).

In this review we will provide an overview of humoral responses to natural infection with AAV and to therapeutic AAV vectors in small and large animal models including humans. We will also discuss the best method to detect these antibody responses and summarize strategies that have been proposed to avoid or overcome NAbs to allow for AAV gene therapy in a wide spectrum of subjects or to patients that already received AAV-mediated gene therapy but need to be re-treated.

## Detection of Anti-AAV Antibodies

Several methods have been developed to detect antibodies to different AAV serotypes. Some of these methods detect total binding antibodies to AAV capsid and other methods detect antibodies that neutralize *in vitro* or *in vivo* transduction of AAV vectors. The first reports in the early 1970s evaluated total antibodies responses to AAV vector as measured by ELISA and Western blot (19–24). These studies focused on AAV1 and AAV2, as these were the AAV serotypes available at that time. The development in the last decade of new AAV types as delivery vectors for gene therapy required more sophisticated assays to evaluate the level of not only binding but NAbs specific to each AAV serotype.

The *in vitro* transduction inhibition assay became the standard assay to evaluate these NAbs to AAV. The assay is usually carried out in a 48- or a 96-well plate format allowing a high throughput sample analysis. Several cell lines have been used as targets for AAV vector transduction: HeLa, 2V6.11, 293, and Huh7 (25–29). Typically an AAV vector expressing a reporter gene is mixed with serial dilutions of the test sample and the vector-serum mixture is incubated with the cell line of choice that is subsequently analyzed for reporter gene expression. The starting dilution of the test sample, which defines the sensitivity of the assay, varies between studies ranging from 1/2 to 1/20 (29–33). In some cases pre-infection of target cells with wild type adenovirus is included to increase AAV transduction. At the same time the AAV vector expressing the reporter gene is mixed with serial dilutions of naïve serum. The purpose of incubating the vector with naïve serum is to evaluate the enhancement in transduction observed at high concentration of serum ≤1/5 (18). The NAb50 titer is reported as the highest serum dilution that inhibits transduction by 50%. *In vitro* transduction efficiency is AAV serotype dependent. The high level of *in vitro* transduction observed with AAV2 combined with the use of very sensitive luminescence reporter genes allows the use of an AAV particle/cell ratio as low as 100 (18). On the other hand, the low levels of *in vitro* transduction observed in some of the new AAV serotypes, like AAV8, demands the use of a higher AAV particle/ratio which for AAV8 is between 1 × 10^3^ and 1 × 10^4^ (10, 18, 27, 30). The use of the minimum amount of AAV particles per cell for each AAV serotype allows the determination of the AAV NAb titer using the most sensitive assay but compromises the comparison of NAb titers between all AAV serotypes. Moreover, the high number of AAV particles/cell in addition to the full/empty AAV particle ratio of the AAV vector preparation used compromises sensitivity and the reproducibility of the results obtained between different laboratories. It should be noted that this assay does not consider the potential antibody-dependent cellular phagocytosis (20) as a blocking mechanism of vector neutralization or the possible change in tissue targeting of AAV (18).

To overcome the above mentioned problems, an *in vivo* NAb assay has been developed by various investigators (10, 18, 34). In this assay, mice are infused with the serum sample immediately prior to intravenous infusion of the AAV vector expressing a secreted reporter gene. Since the level of transduction by the new AAV serotypes is superior to AAV2 in most *in vivo* applications a dose as low as 10^9^ GC/animal can be used. Several secreted transgenes have been used to evaluate transduction including FIX and α-galactosidase (10, 18). A reduction in transgene expression of 50% or more when compared to control mice injected only with AAV vector is reported as positive for AAV NAbs. This assay is more sensitive than the *in vitro* assay as up to 29% of samples that were negative by the *in vitro* NAb assay scored positive for AAV NAbs (10, 18). The problem with this assay is that some of the monkeys that tested positive for AAV NAbs did not show a reduction in AAV-mediated gene transfer, suggesting the *in vivo* NAb assay is too sensitive (18).

The conclusions of these studies suggested that the *in vitro* NAb assay provides a better correlation with *in vivo* transduction in macaques than the *in vivo* NAb assay. Therefore the *in vitro* NAb assay has become the standard assay to evaluate clinical samples for the presence of AAV NAbs prior vector administration.

## Anti-AAV Antibodies in Several Non-Primate Species

Adeno-associated virus has been isolated from several tissues of non-primate animal species including rat, mice, sheep, bird, snake, cows, goat, and pig (2, 7, 24, 35–43), suggesting a natural exposure to AAV. Pre-existing Abs to AAV in these non-primate species were not thought to be a problem because endogenous parvoviruses were believed to be structurally distinct from primate AAVs. Analysis of serum from small and large non-primate species used as pre-clinical animal models has shown high rates of detectable levels of NAbs to several AAV serotypes found in both monkeys and human. Interestingly the prevalence of AAV NAbs is both AAV serotype and species specific (44). In horses, AAV5 is the most seroprevalent serotype with 100% of the samples testing positive for NAbs. In dogs, AAV6 is the most seroprevalent serotype with 100% of the samples positive for NAbs (28, 45). Interestingly, high levels of AAV6 NAbs are found in newborn puppies suggesting passive immunization from colostrum and breast milk (45). A limitation of this study is that the NAb analysis was performed in the same dog breed and colony and no confirmation of AAV6-specific antibodies by ELISA or Western blot was performed. Rapti et al. (28), using mainly pooled sera also found high levels of NAbs to AAV6. In this study, IgG was purified from pooled sera and blocking of AAV transduction was demonstrated, although at much lower titer than whole serum, suggesting that some other factors may play a role in AAV neutralization. A recent study, also in dogs, showed the high binding capacity of serum protein human galectin 3 binding protein to AAV6 inducing the formation of aggregates and hampering vector transduction (46). In pigs, AAV5 is the most seroprevalent AAV serotype with 100% of the samples testing positive for NAbs; seroprevalence of NAbs to other serotypes like AAV1, AAV2, and AAV8 was close to 50%. AAV6 was the least seroprevalent serotype in pigs. *In vivo* testing of serum from these species confirmed the neutralizing activity of these antibodies (28, 44). These studies highlighted the importance of AAV NAb screening of all animal models used to evaluate *in vivo* AAV performance for pre-clinical gene therapy.

## Anti-AAV Antibodies in Primates and Humans

Prevalence of antibodies to AAV in humans was first reported in early 60s and 70s and mainly focused on AAV1 and AAV2, the only serotypes available at that time. Frequencies of antibodies ranged from 30 to 80% among human populations (19, 21–23). Recently, more than 100 natural AAV variants have been isolated from human and non-human primates tissue specimens (2, 6, 41). In pre-clinical models AAV7, AAV8, AAV9, and AAVrh.10 have emerged as promising candidates for gene therapy quickly becoming the most commonly used AAV serotypes in pre-clinical research. Several studies addressed the prevalence of NAbs to these new serotypes and compared them to previously described serotypes (25, 29, 30, 32, 47). In all human populations studied, which included samples from 10 countries and four continents (America, Europe, Africa, and Australia), prevalence of NAbs to AAV2 ranged from 60 to 30% and was significantly higher than the prevalence of NAbs to AAV7, AAV8, and AAV9 serotypes which ranged from 30 to 15%. Although the seroprevalence of NAbs to AAV1 was lower than that for AAV2, it was still higher than AAV7, AAV8, and AAV9 in most regions. It is worth noting that significantly higher frequencies of NAbs to all AAV serotypes were observed in Africa (25). Interestingly, the closely related AAV4 and AAVrh32.33 serotypes showed the lowest seroprevalence with less than 2% of the population testing positive worldwide (25, 35). Although these properties were encouraging for the development of AAVrh32.33 as vector for gene therapy, recent studies have shown this serotype induces a remarkable strong T cell mediated immune responses to the transgene product, similar to that induced by adenovirus, considerably limiting gene expression duration (48). Interestingly, when the seroprevalence to AAV serotypes was analyzed in non-human primates, including rhesus macaques, cynomolgus macaques, Japanese macaques, pig tail macaques, squirrel monkeys, chimpanzees, and baboons, AAV7, AAV8, AAV9, and AAVrh10 were the serotypes with the highest seroprevalence, with NAb frequencies up to 100% (6, 49, 50). Contrary to the situation in humans, AAV2 became the AAV serotype with the lowest seroprevalence (51). This finding is supported by the fact that AAV7, AAV8, and AAVrh10 were originally isolated from rhesus macaque tissues (2, 7). The high seroprevalence of these AAV serotypes in non-human primates presents an important challenge in the evaluation of vector performance in this animal model and an exhaustive screening of monkey colonies for pre-existing NAbs is required.

Binding AAV antibodies may also play an important role in the clinical application of AAV vectors. AAV particles opsonized by non-NAbs may be taken up by cells of the immune system such as dendritic cells and macrophages through Fc receptors which may lead to the development of inflammatory responses. Frequencies of binding antibodies in humans were close to 70% for both AAV1 and AAV2, 45% to both AAV6 and AAV9, and 38% for AAV8 (30). Although the frequencies to binding antibodies were higher than frequencies of NAbs, the relative frequency between AAV serotypes remained the same with AAV1 and AAV2 being the most prevalent AAV serotypes.

Several studies have tried to associate binding antibodies to NAbs with the goal of using binding antibodies as an indirect measure of NAbs (10, 18). Although these studies found a correlation between both types of antibodies, almost 20% of the samples with high binding antibodies had very low or no detectable NAbs. The significance of the Fc interactions to host cells/proteins has been demonstrated in a large animal study in which AAV8 was administered systemically in a non-human primate model with pre-existing AAV8 antibodies. The study showed that AAV vectors were redirected from the liver to the spleen where they were stably sequestered by follicular dendritic cells (18). Although pre-existing humoral immune responses to the AAV capsid do not always correlate with the presence of AAV capsid deposition in the spleen this finding raised concerns of the safety profile of systemic AAV administration.

### Cross-reactivity of the antibody response

An important feature of the humoral response against AAV is the breadth of the response. If a subject is positive for antibodies to a specific AAV serotype, what is the likelihood that this subject will also be positive for other AAV serotypes? Several studies have analyzed the specificity of this response and found a strong link in seropositivity toward distinct AAV serotypes. The majority of the subjects with NAbs and/or binding antibodies to AAV7, AAV8, and AAV9 also had antibodies to AAV2. Conversely, only a few subjects with NAbs and/or binding antibodies to AAV2 had also antibodies to AAV7, AAV8, and AAV9 (25, 30).

Most subjects enrolled in the recent phase 2 clinical trial of AAV1 vector expressing α1-antitrypsin developed a NAb response specific to AAV1 with minimal or no cross-reactivity to AAV2, AAV7, and AAV8 serotypes (15, 52). Only those subjects with low pre-existing NAbs to AAV1, AAV2, AAV7, and AAV8 developed a highly cross-reactive NAb response to AAV2, AAV7, and AAV8 when injected with AAV1. One hypothesis to explain the difference in the breath of the AAV NAb response between naturally exposed and gene therapy-treated subjects is that subjects with co-occurrence of NAbs against multiple AAV serotypes may be the result of multiple infections with various AAV types. Moreover the propensity of AAVs to evolve through various mechanisms of molecular evolution (6) would lead to the generation of NAbs directed against a wide spectrum of homologous antigens. The fact that AAV2 is the serotype with the highest prevalence in the human population and also with the highest NAb titer indicates that the initial and most frequent exposure to AAV in humans occurs with an AAV2 or an AAV2 like serotype (31).

This data would also indicate that subjects naive for AAV NAbs would be the preferred candidates for gene therapy if a second administration of AAV vector is required. These subjects may develop a NAb response specific to the first AAV serotype injected, and a second administration with a different AAV serotype should not pose a problem because of the narrow breath of the AAV NAb response generated. Subjects with pre-existing NAbs to multiple serotypes with a AAV NAb titer low enough (≤1/10) not to interfere with the first vector administration would develop a strong and broadly cross-reactive AAV NAb response that may block a second vector administration with another AAV serotype.

### Age, gender, health status, and geographical region dependent prevalence of NAbs

When and how humans are exposed to AAV for the first time is still not clear, although several studies suggest that this may happen early in life as serum-circulating binding and NAbs have been reported in children (44, 53). This is especially important for the treatment of many genetic diseases that manifest early in infancy and therefore early gene therapy treatment with AAV is beneficial. NAbs to AAV2 were detected in almost 60% of the infants and to AAV8 in 36% of the infants right after birth. Prevalence of NAbs to both AAV serotypes declined during the first year of life due to the drop in maternal antibody levels. A continuous increase in the prevalence of AAV NAbs after 1 year of age with a peak at 3 years of age suggests this age window is the time of the first exposure to natural AAV infections, and closely models that of adenovirus infection (22). Therefore the best age for an early gene therapy intervention with an AAV vector may be right prior to 1 year of age. The higher prevalence of AAV2 NAbs over AAV8 in early childhood also confirms the hypothesis that AAV2 or an AAV2 like serotype is the first AAV to which humans are exposed.

Prevalence of AAV NAbs can vary depending on the geographical origin of the population studied. While the prevalence of AAV1 NAbs in Africa and China is close to 50–70%, in other countries like Belgium, Greece, Italy, and USA it is only 20–30% (25, 54). Overall the prevalence of AAV NAbs seems to be higher in developing countries. It remains unclear whether living conditions, population density, hygienic conditions, different level of health care, MHC background, or method of detecting AAV NAbs are involved in this phenomenon. Interestingly, gender is another factor that influences the prevalence of AAV NAbs. Women have a significantly higher prevalence of AAV1 NAbs than men (54). The health status of the target population may also impact the prevalence of AAV NAbs, especially in those subjects with a compromised immune system. These subjects had a lower prevalence of AAV NAbs when compared to the healthy population (32, 55).

## Strategies to Overcome AAV NAbs in AAV-Mediated Gene Therapy

The presence of AAV NAbs in both animals and human subjects has necessitated the development of strategies to generate new AAV variants with limited or reduced recognition by NAbs. One approach has focused on the identification of the immunogenic domains on the AAV capsid and their subsequent modification to avoid recognition by NAbs. Such targeted engineering of AAV capsid requires knowledge of the antibody recognition site, the epitope. At present, only epitopes to AAV2 and AAV8 serotypes have been identified (56, 57). In this regard, it should be noted that strategies to determine epitopes that are conformational are likely to be more challenging. In spite of these difficulties, it has been demonstrated that amino acid substitutions at sites 459, 493, 551, and 587 of AAV2 capsid confer escape from neutralizing human antisera. While modification of these epitopes provided a reduction in neutralization, it did not completely avoid it. Naturally occurring variants with different amino acid composition at these sites outperformed these mutants in terms of eluding NAbs (8).

An alternate approach is the development of an AAV capsid library generated by error-prone PCR that is subsequently screened in the presence of NAbs to select AAV variants resistant to neutralization. This technique, called directed evolution of AAV vectors, has been employed by Maheshri et al. to identify an AAV2 variant that carried the E12A, K258N, T567S, N587I, and T716A mutations which was 96-fold more resistant to neutralization by anti-AAV2 polyclonal serum than wild type AAV2 (9). A similar approach, but using human serum positive for AAV2 NAbs, identified an AAV2 variant containing R459K and N551D point mutations which was 5.5-fold more resistant to neutralization than wild type AAV2 (58).

The creation of these new AAV variants and the discovery new AAV serotypes (2, 7) with impressive transduction capabilities and distinct serological properties have provided scientists with the tools necessary to avoid undesirable NAbs to one AAV serotype by using a different AAV vector type. This approach has been shown to be effective in both small and large animal models (16, 59). Although this approach is viable for subjects with an AAV NAb response restricted to one serotype or with a cross-NAb titer equal or less than 1/10 (18) it may not be in those subjects with high titers of cross-reactive NAbs. To overcome this problem several strategies have been proposed:

(a) *Plasmapheresis*. Some investigators have explored the use of plasmapheresis to reduce the overall levels of AAV NAbs right before vector administration (10, 27). In this technique, blood is removed and separated into plasma and blood cells. Blood cells are returned to the body and plasma is disposed. An albumin solution is infused into the patient to replace the plasma volume removed (60). Studies using this technique have shown a two to threefold reduction in AAV binding and NAb titers after each sequential apheresis session (10, 18, 27). Although this approach may be very useful for subjects with low levels of broadly cross-reactive AAV NAbs it may still not be enough for those subjects with high levels of NAbs.
(b) Minimizing contact of AAV vector with NAb. Intravenous administration of AAV to target the liver exposes the vector to NAbs present in blood reducing significantly transgene copy number in liver resulting in undetectable levels of transgene expression (10, 11, 18). An approach focused on delivering the vector in the target organ and minimizing the exposure of the vector to circulating NAbs has been shown to be efficient with AAV8 in non-human primate studies targeting the liver via the portal vein (26). In this study investigators flushed the liver with saline, to remove blood and NAbs with it, before injection of the vector into the portal vein. Their results showed no significant impact on gene expression when animals had an AAV NAb titer of up to 1/28 and only partial reduction of gene transfer when the NAb titer was 1/56.
  Another approach to minimize the contact of AAV vector with NAb includes targeting a tissue or organ via direct infiltration rather than via the circulation. This organ/tissue should have a proven record of stable and long term AAV-mediated gene expression. Muscle has been one of the candidate sites to test this approach. Investigators using non-human primates and an AAV8 vector have shown minimal impact on systemic gene expression in the presence of AAV8 NAbs as high as 1/320 when monkeys were injected directly into muscle (61). A similar approach has been used in clinical trials for A1AT deficiency and hemophilia B using AAV1 and AAV2 vectors respectively, demonstrating that high titers of AAV NAb did not prevent gene transfer (15, 52, 62). The retina has also been used as a target organ for AAV-mediated gene therapy for localized systemic expression of therapeutic proteins such us erythropoietin (63). As into the muscle, the subretinal space into which the vector is injected has reduced contact with blood; consequently pre-existing AAV NAbs in blood had a minimal impact of AAV-mediated gene transfer (64, 65). Intrathecal administration (i.e., direct injection into the cerebral spinal fluid) is another route of administration that minimizes contact of the AAV vector with blood. In recent mouse studies, AAV2 and AAV5 were injected intrathecally to transduce the central nervous system (66). These studies showed that the potentially debilitating effect of AAV NAbs could be partially overcome by direct administration of AAV to the target organ. The caveat associated with these alternative routes of administration is the immune response that is induced in these organs/tissues. For example, the muscle is a very immunogenic tissue and intramuscular administration, unlike intravenous administration, induces a strong humoral and cellular immune response to the AAV capsid and in some instances to the transgene product (15). Humoral and cellular immune responses to the AAV capsid and transgene product using these alternative routes of administration need to be carefully assessed to ensure that the lack of AAV interference with pre-existing NAbs is not eclipsed by the induction of a stronger immune response to the therapeutic product.
(c) Immunosuppression has been proposed by several groups as a method to lowering NAbs by reducing the number of cells producing antibodies. In these studies rituximab, a B-cell depleting antibody that targets the CD20 antigen and is used clinically, was able to reduce AAV2 and AAV5 NAbs in ∼30% of subjects to levels that for some subjects were under limit of detection (32). These data are consistent with results obtained in non-human primates using rituximab and cyclosporine, although in this study rhesus macaques were injected systemically with an AAV vector and then NAb response were monitored (67). The caveat with pharmacological immunomodulation of the immune response is that the tolerogenic properties of AAV can be altered after this treatment and undesirable immune response to the transgene may be induced, as was reported for monkeys injected with AAV2 and carrying FIX as transgene (68). An immediate antibody response to the FIX was observed when the immunosuppression regimen was stopped after 10 weeks of vector administration. An antibody response to FIX was never observed in monkeys that did not receive immunosuppression.

In conclusion, a combination of alternative AAV types, route of vector administration with minimum contact with blood and techniques directed to lower AAV NAb by physical methods or pharmacological modulation of the humoral immune response may ultimately overcome the impact of pre-existing AAV NAbs in subjects who would otherwise not be eligible for AAV-mediated gene transfer therapy.

## Conflict of Interest Statement

James M. Wilson is a consultant to ReGenX Holdings and is a founder of, holds equity in, and receives a grant from affiliates of ReGenX Holdings; in addition, relevant to this work, he is an inventor on patents licensed to various biopharmaceutical companies, including affiliates of ReGenX Holdings. Roberto Calcedo declares no competing interests. James M. Wilson holds a patent on adeno-associated virus (AAV) clades (U.S. Patent 7,906,111B2) with pending continuation (U.S. Patent 13/023,918).
